# Changes to mitochondrial ultrastructure in optic nerve vulnerable to secondary degeneration in vivo are limited by irradiation at 670 nm

**DOI:** 10.1186/1471-2202-14-98

**Published:** 2013-09-08

**Authors:** Nadia Cummins, Carole A Bartlett, Michael Archer, Elora Bartlett, Jan M Hemmi, Alan R Harvey, Sarah A Dunlop, Melinda Fitzgerald

**Affiliations:** 1 Experimental and Regenerative Neurosciences, The University of Western Australia, Crawley 6009, WA, Australia; 2 School of Animal Biology, The University of Western Australia, Crawley 6009, WA, Australia; 3 UWA Oceans Institute, The University of Western Australia, Crawley 6009, WA, Australia; 4 School of Anatomy, Physiology and Human Biology, The University of Western Australia, Crawley 6009, WA, Australia

## Abstract

**Background:**

Traumatic injury to the central nervous system results in damage to tissue beyond the primary injury, termed secondary degeneration. Key events thought to be associated with secondary degeneration involve aspects of mitochondrial function which may be modulated by red/near-infrared irradiation therapy (R/NIR-IT), but precisely how mitochondria are affected *in vivo* has not been investigated. Secondary degeneration was modelled by transecting the dorsal aspect of the optic nerve in adult rats and mitochondrial ultrastructure in intact ventral optic nerve vulnerable to secondary degeneration investigated with transmission electron microscopy.

**Results:**

Despite reported increases in fission following central nervous system injury, we saw no change in mitochondrial densities in optic nerve vulnerable to secondary degeneration *in vivo*. However, in axons, frequency distributions of mitochondrial profile areas showed higher cumulative probabilities of smaller mitochondrial profiles at day 1 after injury. Glial mitochondrial profiles did not exhibit changes in area, but a more elliptical mitochondrial shape was observed at both day 1 and 7 following injury. Importantly, mitochondrial autophagic profiles were observed at days 1 and 7 in optic nerve vulnerable to secondary degeneration *in vivo*. Citrate synthase activity was used as an additional measure of mitochondrial mass in ventral optic nerve and was decreased at day 7, whereas mitochondrial aconitase activity increased at day 1 and day 28 after injury in optic nerve vulnerable to secondary degeneration. R/NIR-IT has been used to treat the injured central nervous system, with reported improvements in oxidative metabolism suggesting mitochondrial involvement, but ultrastructural information is lacking. Here we show that R/NIR-IT of injured animals resulted in distributions of mitochondrial areas and shape not significantly different from control and significantly reduced mitochondrial autophagic profiles. R/NIR-IT also resulted in decreased citrate synthase activity (day 7) and increased aconitase activity (day 1) in optic nerve vulnerable to secondary degeneration.

**Conclusions:**

These findings suggest that mitochondrial structure and activity of enzymes of the citric acid cycle are dynamically altered during secondary degeneration *in vivo* and R/NIR-IT may protect mitochondrial structure.

## Background

Acute injury to the central nervous system (CNS) is associated with secondary degeneration involving both neurons and glia neighbouring the lesion, resulting in further functional loss [1,2]. Mitochondrial dysfunction is a critical component of CNS injury [3]. Intracellular Ca^2+^ concentrations are disturbed both at and adjacent to the injury [4], and mitochondria take up excess Ca^2+^ to maintain homeostasis in the cytosol [5]. However, excess mitochondrial Ca^2+^ may lead to the opening of the mitochondrial permeability transition pore, with resultant impairment in mitochondrial function and metabolic activity and/or cell death [6]. Further, mitochondria are a major source of reactive oxygen species (ROS), the production of which is increased with elevated mitochondrial Ca^2+^ concentrations [7]. We have previously demonstrated both altered Ca^2+^ distributions and oxidative stress in white matter vulnerable to secondary degeneration [8].

Mitochondria are able to respond to the changing energy needs of the cell, as well as perform their own damage control, through their ability to divide (fission) or fuse [9]. These processes are mediated by GTPases [10] and are partly regulated by Ca^2+^ levels, with influx of Ca^2+^ through voltage-gated Ca^2+^ channels causing increased mitochondrial fission *in vitro*[11]. Impairments in the balance between fission and fusion can lead to disruptions in mitochondrial motility, ATP generation and Ca^2+^ buffering. Such changes have been described in a number of pathological situations, including neurodegenerative disorders, stroke models and conditions of oxidative stress [12], but mitochondrial densities and ultrastructure have not yet been assessed *in vivo* in white matter vulnerable to secondary degeneration.

It was hypothesised that the balance of mitochondrial fission/fusion would alter as a feature of secondary degeneration *in vivo*. Accordingly, using the partial optic nerve (ON) transection model of secondary degeneration [13,14], we assessed density of mitochondrial profiles to analyse possible changes *in vivo*[15]. Further, area and shape of mitochondrial profiles were assessed, as these aspects of mitochondrial structure have been shown to be related to osmotic conditions, altered mitochondrial function and may also reflect changes associated with fission and/or fusion [16]. Citrate synthase activity is widely used as an indicator of mitochondrial mass [17] and was assessed here as an adjunct to the ultrastructural analyses, together with mitochondrial aconitase activity, which has been shown to be susceptible to certain reactive species [18] that may contribute to secondary degeneration.

Red/near-infrared irradiation therapy (R/NIR-IT, 630 - 1000 nm) has been used as a safe treatment for a range of CNS disorders and injuries [19-21] and is currently being assessed in clinical trials for stroke [22]. The mitochondrial enzyme cytochrome c oxidase has been posited as the most likely photoacceptor for R/NIR-IT [23,24], and treatment is associated with improvements in oxidative metabolism, including increased ATP production [19,24,25]. We have demonstrated that R/NIR-IT results in reduced oxidative stress and preservation of function in ON vulnerable to secondary degeneration [26]. However, the effects of R/NIR-IT on mitochondrial ultrastructure and citric acid cycle enzyme activities in CNS injury and secondary degeneration *in vivo* have not yet been assessed. Here we show that mitochondria exhibit subtle ultrastructural changes during secondary degeneration, as well as an increased appearance of mitochondrial autophagic profiles. Following R/NIR-IT, the mitochondrial area and shape changes seen during secondary degeneration were no longer observed, and mitochondrial autophagic profiles were reduced.

## Results

### Density of mitochondrial profiles did not change in areas of ON vulnerable to secondary degeneration +/− R/NIR-IT

**Figure 1:**
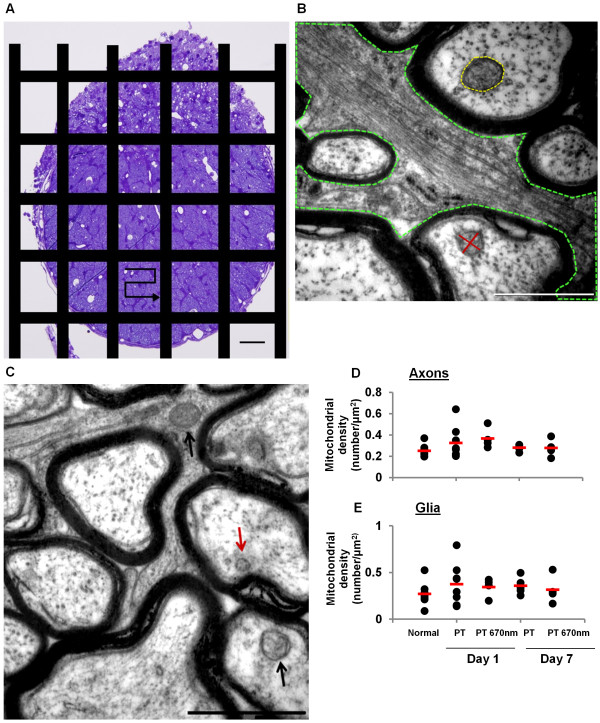
**Density of mitochondrial profiles in ON vulnerable to secondary degeneration +/− R/NIR-IT. (A)** Transverse section (semi-thin) of an ON lesion site, stained with 0.5% toluidine blue. The black bars are a schematic of the copper grid overlaying ultrathin sections and the arrow represents the path taken during sampling from one ventral region, bounded by the grid; scale = 50 μm. **(B)** Representative electron micrograph illustrating definition of axonal and glial cellular compartments, tracing of glial processes is shown in green. Diameters of mitochondria were measured at the longest extent and a second measurement perpendicular to the first through the midline was made for assessment of mitochondrial ellipticity (red). Area was measured by manually tracing around the outer membrane (yellow); scale = 1 μm. **(C)** Representative images illustrating criteria for classification as mitochondria ie: double membrane, increased electron density and/ or convolutions (black arrows) or excluded due to lack of double membrane (red arrow); scale = 1 μm. Mean densities of mitochondrial profiles did not change with injury or R/NIR-IT in axons **(D)** or glia **(E)**, p >0.05. Black dots represent means for each animal and the red line indicates the group mean.

Mitochondria can respond to stress in part by altering the balance between fission and fusion [15]. It was hypothesised that such an alteration would be reflected in a change of mitochondrial numbers following injury, indicated by the number of mitochondrial profiles in TEM images from transverse ON sections [27]. We assessed mitochondrial profiles at 1 and 7 days after injury, as we have already demonstrated that the oxidative stress response is well established at 1 day [1] and mitochondrial ultrastructure changes as a response to oxidative stress may be beginning to resolve at 7 days. The densities of mitochondrial profiles were separately quantified for axonal and glial cellular compartments. Axons were clearly identified by the surrounding electron dense myelin sheath and glial compartments were defined as the non-axonal areas within the image (Figure 1B). Mitochondria were identified on the basis of a clear double membrane, increased electron density and/or the presence of convolutions (Figure 1C). It was not necessary to correct density values for ventral ON area as there was no change in the area of the ventral region observed with injury, at the time points assessed in the current study (ANOVA, F = 0.1, p = 0.87, data not shown). Furthermore, no significant differences between control groups were detected (unhandled vs handled animals) for any of the TEM outcomes (all p > 0.05); therefore data from these groups were pooled. There were no significant changes in mean mitochondrial density with injury or R/NIR-IT, in either axonal or glial cellular compartments (Figure 1D, E; F = 1.2, p = 0.32 and F = 0.54, p = 0.71, respectively). This implies that mitochondrial fission was not a feature of secondary degeneration in this model, at 1 and 7 days after injury.

### Area and shape changes of mitochondrial profiles in ON vulnerable to secondary degeneration +/− R/NIR-IT

**Figure 2:**
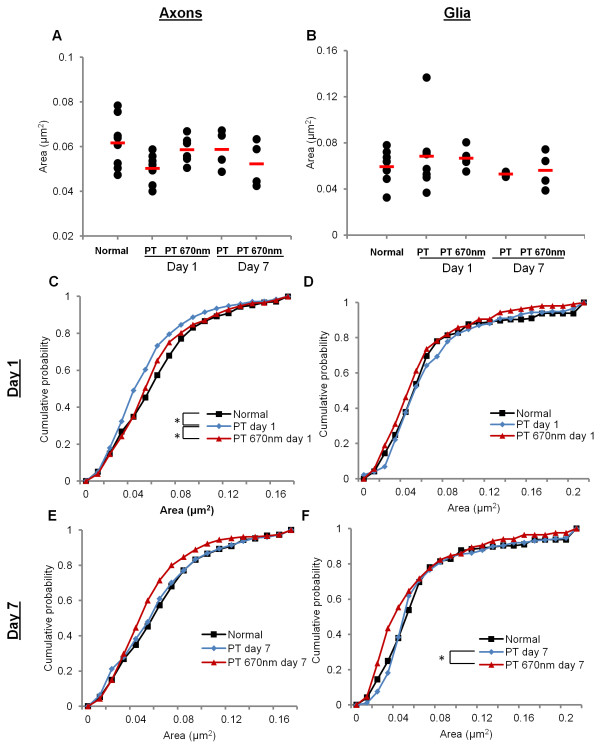
**Changes in mitochondrial profile cross-sectional area in secondary degeneration +/− R/NIR-IT.** Mean area did not change with either injury or R/NIR-IT in axons **(A)** or glia **(B)**. At day 1 after PT, mitochondrial profiles with smaller areas became more frequent in axons, and this was no longer observed following R/NIR-IT at this time **(C)**. There were no changes in the distributions of glial mitochondria at day 1 **(D)**. At day 7, there was no change in the distribution of area of axonal mitochondrial profiles **(E)**. R/NIR-IT resulted in an increased cumulative probability of mitochondria with smaller cross-sectional areas compared to PT day 7 in glia, however neither the distributions of injured animals (PT) or R/NIR-IT animals (PT 670 nm) were different from normal **(F)**. *Significantly different at p ≤ 0.05.

Aspects of mitochondrial structure, including size and shape, are related to function [16]. We therefore assessed cross-sectional area of mitochondrial profiles (Figure 1B) in order to detect changes occurring as a consequence of secondary degeneration. Mean area of mitochondrial profiles was not significantly different following PT injury or R/NIR-IT in either axons (Figure 2A, F = 2.1, p = 0.11) or glia (Figure 2B, F = 0.69, p = 0.60). However, comparison of frequency distributions of mitochondrial area (displayed as cumulative probabilities) revealed subtle differences not apparent in means comparisons. At day 1 after PT, there was a significant change in the distribution of areas of axonal mitochondrial profiles in ventral ON vulnerable to secondary degeneration (Figure 2C, d = 0.17, p = 0.02), with an increase in the cumulative probability of smaller mitochondria following injury. No significant change was observed at day 7 compared to normal in axonal mitochondria (Figure 2E, d = 0.07, p = 0.61). Following R/NIR-IT, the frequency distribution of areas of axonal mitochondrial profiles was no longer significantly different from normal animals (d = 0.09, p = 0.45), and there was a shift in the cumulative probabilities towards larger mitochondria compared to injured animals (d = 0.16, p = 0.03). There was no statistically significant effect of R/NIR-IT on the distribution of axonal mitochondrial area at day 7 (d = 0.14, p = 0.15). Mitochondria are generally longitudinally aligned along the ON [28]. Therefore the lengths of axonal mitochondria were also assessed in longitudinal sections from a separate cohort of animals. Pilot data indicated that mitochondrial length did not change at 1 day after injury in ON vulnerable to secondary degeneration (mean ± S.E.M: normal = 0.98 ± 0.10 μm; PT day 1 = 0.82 ± 0.05 μm; t = 1.40, p = 0.23).

**Figure 3:**
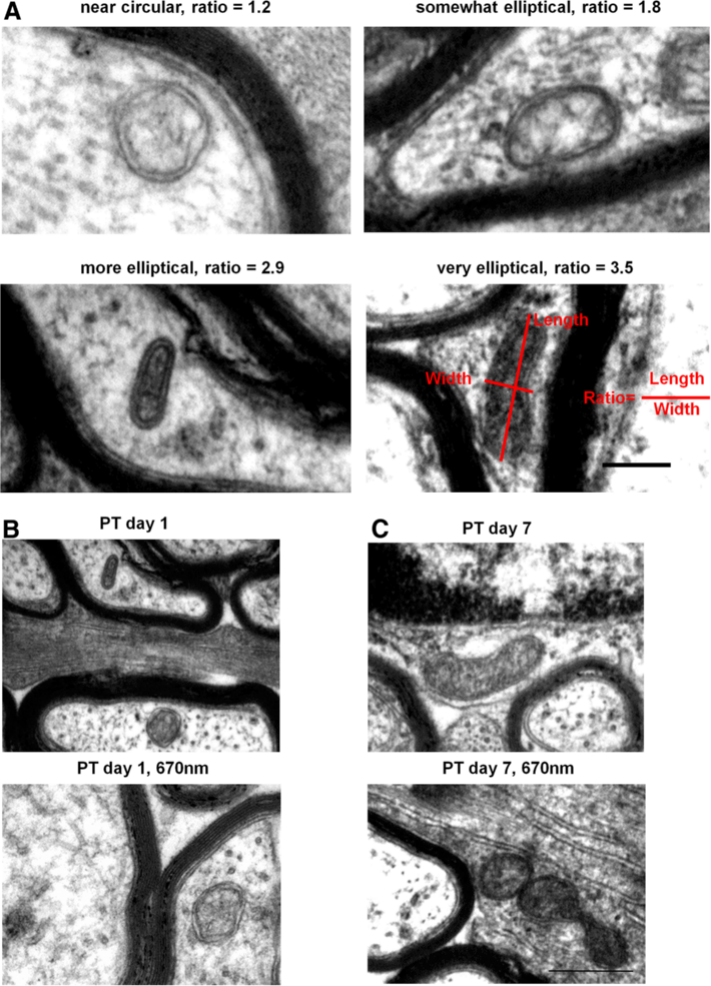
**Mitochondrial shape changes in secondary degeneration +/− R/NIR-IT.** Representative images of shapes represented by common ratios, scale = 200 nm **(A)**. Representative images illustrating lack of change in mitochondrial profile shape with R/NIR-IT at day 1 in axons **(B)** and more circular mitochondrial profiles with R/NIR-IT at day 7 in glia **(C)**; scales = 500 nm.

In glia, no significant change in the distribution of mitochondrial profile areas was observed due to secondary degeneration at day 1 (Figure 2D, d = 0.11, p = 0.40), or at day 7 (Figure 2F, d = 0.09, p = 0.43). Despite this, and in contrast to effects observed in axonal compartments, R/NIR-IT resulted in a reduction in glial mitochondrial profile area at day 7 after injury, as reflected by an increase in the cumulative probability of smaller mitochondria in injured animals (d = 0.27, p = 0.01). However, the difference in distribution was not significantly different when compared to normal animals (d = 0.2, p = 0.17) (Figure 3F).

**Figure 4:**
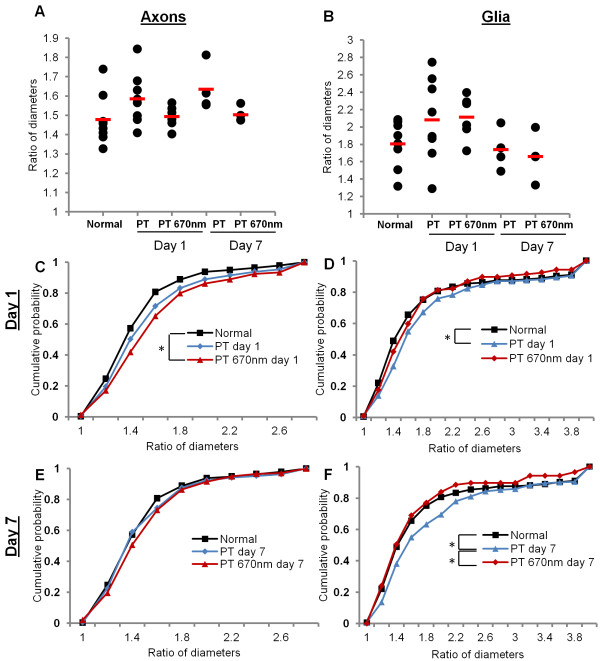
**Quantification of mitochondrial shape changes in secondary degeneration +/- R/NIR-IT**. **Mean ratios of diameters did not change with either injury or R/NIR-IT in axons (A) or glia (B).** At day 1, there was no change in mitochondrial profile shape in axons **(C)**, but a higher probability of more elliptical profiles (signified by larger ratios) was observed in glia **(D)**. Following R/NIR-IT, the distributions of mitochondrial profile shape in glia were not significantly different from normal. At day 7 following injury, the distribution of mitochondrial profile shape was not different compared to normal in axons **(E)**, but again a higher cumulative probability of more elliptical profiles in glia was observed **(F)**; a change which was no longer detected after R/NIR-IT. *Significantly different at p ≤ 0.05.

For every mitochondrial profile assessed, a ratio between the minimum and maximum diameter was generated (Figure 1B, 3A), providing a quantitative assessment of mitochondrial profile shape. Ratios closer to 1 reflect more circular profiles, while larger ratios represent more elliptical shapes. Representative images of mitochondria exhibiting different ratios are displayed in Figure 3A. Similarly to analyses of mitochondrial profile area, there were no differences in mean mitochondrial profile shape following PT injury or R/NIR-IT in axons (Figure 4A, F = 2.01, p = 0.12) or glia (Figure 4B, F = 0.12, p = 0.13). Analyses of frequency distributions of mitochondrial profile shape showed that while there was a trend towards more elliptical profiles, there were no statistically significant changes in the distributions of mitochondrial profile shape in secondary degeneration in axons at either day 1 (Figure 4C, d = 0.10, p = 0.06) or day 7 (Figure 4E, d = 0.07, p = 0.41). While R/NIR-IT had no effect on these distributions when compared to injured animals in axons at day 1 (Figure 4C, E, Figure 3B; d = 0.11, p = 0.08) or day 7 (d = 0.10, p = 0.15), distributions indicated an increasingly elliptical shape compared to normal animals at day 1 (d = 0.17, p = 0.02) but not at day 7 (d = 0.10, p = 0.18).

In glia, the distribution of mitochondrial profile shapes was significantly altered towards a higher probability of more elliptical profiles (Figure 4D, d = 0.20, p = 0.04), and this was also observed at day 7 (Figure 4F, d = 0.17, p = 0.03). Following R/NIR-IT, the distribution of mitochondrial profile shapes was significantly less elliptical compared to the untreated group at 7 days after injury (Figure 4F, 3C; d = 0.18, p = 0.02) and was no longer significantly different from normal at either day 1 (Figure 4D, d = 0.13, p = 0.40) or day 7 (Figure 4F, d = 0.09, p = 0.84). R/NIR-IT did not significantly alter the distribution of mitochondrial profile shape compared to the untreated group at 1 day after injury (d = 0.11, p = 0.58), but distributions were also not different from normal (Figure 4D, d = 0.12, p = 0.40). This finding suggests that R/NIR-IT either prevented the change in mitochondrial profile shape seen in secondary degeneration, or restored normal shape.

### Mitochondrial autophagy in ON vulnerable to secondary degeneration +/− R/NIR-IT

**Figure 5:**
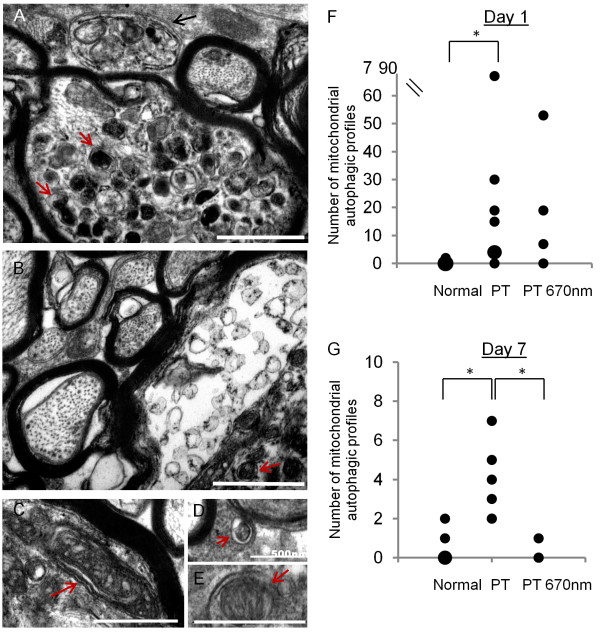
**Mitochondrial autophagic profiles in ON vulnerable to secondary degeneration +/− R/NIR-IT.** Accumulations of mitochondrial autophagic profiles (red arrows) were seen especially at day 1 after PT **(A**, **B)**. Occasionally, larger features reminiscent of autophagosomes were observed **(A**, black arrow**)** and parts of the nerve appeared highly abnormal (*, **B)**. Further representative images of mitochondria (identified by their double membrane) enveloped by an additional double membrane **(C**, **D**, **E**, red arrows, classified as mitochondrial autophagic profiles**)** are shown; scale bar = 1 μm unless otherwise stated on image. Number of mitochondrial autophagic profiles at day 1 **(F)**: each black dot represents one animal, and larger dots represent a higher frequency of that particular number of observations. There were significantly more observations of mitochondrial autophagic profiles at day 1 after PT injury compared to normal, which was not significantly altered by R/NIR-IT. At day 7 there was an increased incidence of mitochondrial autophagic profiles compared to normal and the incidence was decreased following R/NIR-IT **(G)**. Note that for this outcome, no distinction between glia and axons was made, due to the number of mitochondrial autophagic profiles in glia being very low. *Significantly different at p ≤ 0.05.

Ultrastructural analyses of mitochondria revealed a small subset which featured second double membranes, very high electro-density and/ or vacuole-like features, clearly indicative of autophagic processes [29,30], and which precluded their inclusion in density analyses described to this point. The envelopment of mitochondria in autophagic membranes is suggestive of their elimination by mitophagy (Figure 5A-E). However definitive identification of mitophagy (as opposed to other autophagic elements) is hampered by lack of selective markers applicable to both axons and glia [31], the fact that structural features in TEM images can be equivocal and that structural and chemical features of autophagy in tissue sections *in vivo* are yet to be fully characterised [32]. Therefore, these structures were classified as mitochondrial autophagic profiles and their incidence quantified per field of view (30 fields of view/animal, Figure 4F, G). The incidence of mitochondrial autophagic profiles in ventral ON was significantly higher at days 1 and 7 after injury compared to normal (Figure 5F, G, F = 5.53, p < 0.001; F = 34.98, p < 0.001, respectively). Treatment with R/NIR-IT did not affect the incidence of mitochondrial autophagic profiles compared to injured animals at day 1 (F = 0.42, p = 0.74). At day 7 however, R/NIR-IT resulted in a smaller number of mitochondrial autophagic profiles compared to injured animals (Figure 5G, F = 26.73, p < 0.01), with numbers not significantly different from normal (F = 0.09, p = 0.77).

### Aconitase and citrate synthase activities in ON following PT injury +/− R/NIR-IT

**Figure 6:**
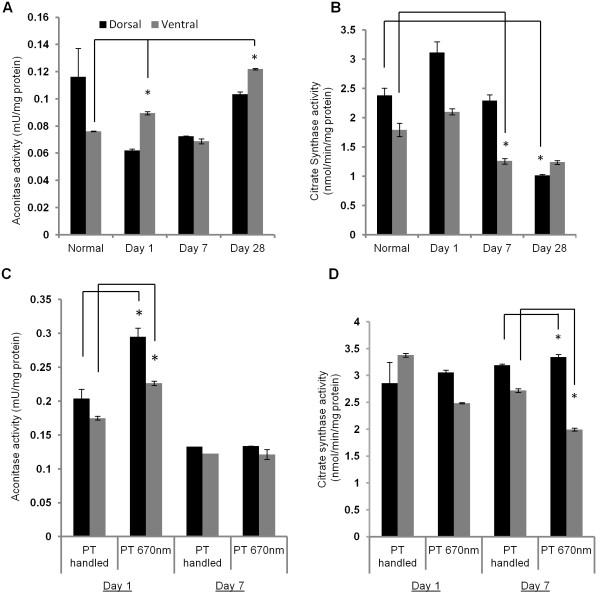
**Aconitase and citrate synthase activities after PT injury +/− R/NIR-IT.** Aconitase activity (mean ± SEM) increased in ventral ON vulnerable to secondary degeneration after PT injury compared to normal **(A)** and with R/NIR-IT compared to PT day 1 in both ventral and dorsal ON **(C)**. Citrate synthase activity (mean ± SEM) decreased after injury compared to normal **(B)** and following R/NIR-IT compared to PT at day 7 **(D)**. *Significantly different at p ≤ 0.05.

The activities of the mitochondrial enzymes aconitase and citrate synthase were assessed in homogenates of both dorsal ON exposed to the primary injury and ventral ON vulnerable to secondary degeneration (n = 6 pooled ONs/group). Aconitase activity has been shown to be inhibited by certain species of ROS [18,33] and as such has been used to indicate altered mitochondrial function due to oxidative stress [34]. Citrate synthase was assessed as an adjunct to the TEM analyses of mitochondrial density and size, to provide an additional marker of mitochondrial content [17,35]. PT injury led to significant differences in mean activity for both aconitase (Figure 6A, V = 1.8, p < 0.001) and citrate synthase (Figure 6B, V = 1.43, p < 0.001). Follow up ANOVAs and Bonferroni *post hoc* tests demonstrated significant increases in aconitase activity compared to normal at days 1 and 28 after injury in ventral ON (F = 424.41, p < 0.001) and significant decreases in citrate synthase activity compared to normal in ventral ON at day 7 (F = 21.94, p < 0.001) and in dorsal ON at day 28 (F = 12.17, p = 0.002). No statistically significant differences in dorsal ON aconitase activity were detected (F = 5.9, p = 0.06), likely due to relatively high S.E.M in the normal group (Figure 6A, B).

Following R/NIR-IT, aconitase activity was increased in both dorsal and ventral ON (V = 0.935 p = 0.02) at day 1 after injury (Figure 6C, F = 24.37, p = 0.01 and F = 36.26, p < 0.01, for dorsal and ventral respectively). R/NIR-IT also affected citrate synthase activity in both dorsal and ventral ON as follows (V = 0.995, p < 0.001); while activity was significantly decreased in ventral ON at day 7 after injury (F = 768.19, p < 0.001) activity was significantly increased in dorsal ON (F =149.55, p < 0.001) (Figure 6D).

## Discussion

We have demonstrated that in ON vulnerable to secondary degeneration, densities of mitochondrial profiles were not altered, indicating no change to the balance of fission and fusion. More detailed analyses of distributions of mitochondrial size and shape revealed subtle alterations during secondary degeneration. Axonal mitochondrial profiles were smaller 1 day following injury, perhaps indicating a tendency towards fission, or alternatively, shrinkage in response to changing osmotic conditions. In contrast, glial mitochondrial profile area did not change, but profiles were more elliptical at days 1 and 7 after PT injury. While the observation of elliptical profiles depends upon the sectioning of the tissue, increased frequency of elliptical profiles together with no change in area indicates that on average, mitochondria in glia were becoming longer and thinner, perhaps indicating fusion. However the lack of increase in density of mitochondrial profiles indicates that these changes were relatively minor and marked alteration in the balance of fission/fusion in ON vulnerable to secondary degeneration at day 1 is unlikely. Our findings are of interest as they are in contrast to reported increases in fission as a consequence of stroke [12], although changes at earlier time points may occur. Importantly, there was a significant increase in the appearance of mitochondrial autophagic profiles at days 1 and 7 in ON vulnerable to secondary degeneration, as has been reported in a range of CNS injury models [29,30], indicating vulnerability of mitochondria to secondary degeneration.

The lack of change in citrate synthase activity in ON at day 1 after injury reflected the unchanged density of mitochondrial profiles at this time, indicating that mitochondrial volume was maintained. However, despite constant mitochondrial density, citrate synthase activity decreased in ventral ON at day 7, perhaps due to the demonstrated inhibition of this enzyme by alkyl peroxyl and alkoxyl radicals [36], which are elevated following traumatic brain injury [37]. In contrast, we observed increases in aconitase activity in ventral ON. Given that we did not observe greater mitochondrial density in ventral ON after injury, we can exclude that the increase in aconitase reflects more mitochondria. Aconitase is known to be inhibited by ROS such as superoxide [38], H_2_O_2_[18], and peroxynitrite [33]. Coupled with our reported increase in MnSOD immunointensity at 1 and 7 days after partial ON transection [1], these data suggest that superoxide levels are effectively controlled by endogenous mechanisms in our model. An approximately 50% increase in aconitase activity has been reported following repeated contractions of mouse skeletal muscle, despite increased ROS production [39], but mechanisms are unclear and further work is required to understand the effects of secondary degeneration on the activity of citric acid cycle enzymes.

R/NIR-IT prevented the subtle decrease in axonal mitochondrial profile size, suggesting that for this outcome, normal mitochondrial ultrastructure was either maintained despite injury, or restored within the first 24 hours with R/NIR-IT. Similarly, following R/NIR-IT in PT injured ON, mitochondrial shape in glia was no longer different from normal. Importantly, the appearance of mitochondrial autophagic profiles observed in ON vulnerable to secondary degeneration was also reduced by R/NIR-IT at day 7. The finding that R/NIR-IT partly prevented the changes in mitochondrial ultrastructure seen in secondary degeneration *in vivo* is in line with previous studies reporting beneficial effects of R/NIR-IT on mitochondrial metabolism associated with functional improvements. These include increased visual pathway and whole-brain metabolism as assessed using cytochrome c oxidase activity and oxygen consumption [20], recovery of rod and cone-mediated function after inhibition of cytochrome c oxidase with formic acid derived from the *in vivo* breakdown of methanol [19], as well as reductions in markers of oxidative stress [26,40]. Our findings suggest that these improvements in oxidative metabolism with R/NIR-IT could be in part due to preservation of normal mitochondrial structure, which goes hand-in-hand with normal mitochondrial function [15,41,42]. By extension, improvements in the structure of mitochondria may explain the improvements in visual function previously reported in our model following R/NIR-IT [26].

Very few studies have examined the effect of R/NIR-IT on mitochondrial morphology, and these have used *in vitro* models of non-CNS systems. In human lymphocytes, irradiation with He-Ne laser (λ_max_ = 632.8 nm) resulted in the formation of giant, branched mitochondria at 1 hour post-treatment [43]. These were shown to be due to fusion of smaller mitochondria and proposed to be more efficient at energy transfer [43]. Giant mitochondria have been described in rat axon hillocks [44], with a characteristic diameter of approximately 1 μm, but were not observed in the present study. Structural changes of the giant mitochondrion normally present in yeast cells after irradiation with He-Ne laser have also been investigated [45] and it was found that eighteen hours after irradiation, the mitochondrial matrix had expanded, providing conditions more favourable to energy transfer in the form of membrane potential. A similar scenario could be responsible for a restoration of axonal mitochondrial profiles to normal size by day 1 in the current model and the increasingly elliptical shape of mitochondria in axons compared to normal at day 1. However, it is also possible that mitochondrial size was simply maintained and/or increased *via* a protective effect due to increased cytochrome c oxidase activity, as has been reported with R/NIR-IT [23,24], prevention of reverse electron transport and maintenance of the mitochondrial permeability transition [7,46] or restoration of normal osmotic conditions. R/NIR-IT also prevented the shift towards more elliptical mitochondrial profiles that was observed in glia at day 1 and 7 in ON vulnerable to secondary degeneration. As a number of pathological circumstances result in a transition from elliptical, rod-like mitochondria to more rounded mitochondria, it is unclear whether the increasingly elliptical shape seen after PT injury reflects functional impairment, or is perhaps a reflection of cytoskeletal changes following injury [47-49]. Similarly, the altered citrate synthase activity and increased aconitase activity observed following R/NIR-IT could reflect impairment as a consequence of irradiation, but the complexities in the responses to injury of these enzymes indicates that more detailed analyses are required to understand these effects and ascertain whether these changes in enzyme activity are more or less important than the observed structural restorations. Further, given the host of studies reporting beneficial effects of R/NIR-IR on mitochondrial function, it is unlikely that the observed alterations in enzyme activities are detrimental [20,24,50]. R/NIR-IT resulted in increased relative matrix surface area in yeast mitochondria [45] and this improves their aerobic capacity [51], however assessment of cristae was beyond the scope of the current study.

## Conclusions

Mitochondria exhibit both structural and functional changes in secondary degeneration, which are likely to play an important role in the pathophysiology of this process. We have provided evidence that R/NIR-IT has potentially beneficial effects on mitochondrial structure in secondary degeneration and can also affect citric acid cycle activity. Understanding the mechanism by which R/NIR-IT exerts its positive effects on function is valuable, as this therapy is both clinically relevant and readily translatable, offering an exciting candidate for treatment of secondary degeneration following injury to the CNS.

## Methods

### Animals and surgery

All experimental procedures conformed to ‘Principles of Laboratory Animal Care’ and were approved by the Animal Ethics Committee of the University of Western Australia. Adult female Piebald Virol Glaxo (PVG) rats (160-200 g) obtained from the Animal Resource Centre (Murdoch, WA) were housed in clear plastic cages with food and water *ad libitum* under a standard 12 hour light/dark cycle. Animals were anaesthetised with an intraperitoneal injection of xylazine (10 mg/kg, Troy Laboratories Pty Ltd) and ketamine (50 mg/kg, Troy Laboratories Pty Ltd) and euthanized with Euthal (active constituent Pentobarbitone Sodium 160 mg/ml, Virbac Australia Pty Ltd). Following anaesthesia, animals received a partial transection (PT) to the right ON, as described previously [14,52]. Briefly, a controlled 200 μm cut to the dorsal surface of the optic nerve was made with a radial keratotomy diamond knife (Geuder), approximately 1 mm behind the eye, with care taken to minimise damage to the lachrymal glands or major blood vessels. The animals recovered on a warming blanket and were rehydrated with a subcutaneous injection of 1 ml phosphate buffered saline (PBS) as well as treated subcutaneously with an analgesic (carprofen, 4.0 mg/kg, Norbrook Australia Pty Ltd).

### R/NIR-IT

R/NIR-IT was administered using a VET 75 LED array (Quantum Devices) positioned 3 cm above and parallel to the dorsal surface of the head of the animal, so that the entire head was irradiated. Light was delivered with a centre wavelength of 670 nm and a full-width at half maximum bandwidth of approximately 22 nm emitting 5 joules of energy per 88-second dose at 60 mW*/* cm^2^ at a total fluence of 4.5 W. The array was air cooled. Choice of treatment time and administration details were based on the literature and beneficial effects previously described following ON injury [20,26,53]. Animals were treated once per day for 30 minutes, with the first treatment administered immediately following PT surgery, while the animal was recovering from anaesthesia, temperature maintained on a 37°C heat-pad. Subsequent treatments involved loosely holding the un-anaesthetised animal under the LED array, minimising stress and preventing rises in body temperature; as measured by rectal thermometer. Animals were euthanized and either perfused or dissected (as described below) immediately after completion of the final treatment session. Control animals (in groups as described below) received sham handling, under a cardboard box of the same dimensions as the LED array.

### Transmission electron microscopy (TEM) assessment of mitochondrial ultrastructure: tissue preparation

Mitochondrial ultrastructure was assessed at two time points post PT (day 1 or day 7). Three groups per time point (n = 3-4/ group) received a PT of the ON, followed by either R/NIR-IT (PT 670 nm), handling (PT handled) or no further intervention (PT). The fourth group was an uninjured, but handled control (Normal handled, day 1 only due to constraints of study size). An additional group of completely normal (unhandled) animals was also assessed. The study design is summarised in Table 1. Animals were euthanized and transcardially perfused with 150 ml 0.9% saline, followed by 150 ml of modified Karnovsky’s fixative (2.5% glutaraldehyde, 1% paraformaldehyde, 0.15 M Sorenson’s Phosphate buffer, 2% sucrose, pH = 7.2). ONs were dissected and stored in Sorenson’s Phosphate buffer until post-fixed in 1% osmium tetroxide (ProSciTech) in 0.15 M phosphate buffer, 1 hr. Nerves were then dehydrated through graded alcohols and propylene oxide before embedding in epoxy resin. Nerves were sectioned in the transverse plane on an Ultrotome NOVA microtome; ultrathin 100 nm sections were cut from the lesion site, collected onto copper grids and stained with uranyl acetate, blot dried and then stained with Reynold’s lead citrate for 2 min. Additional animals were used for pilot studies of mitochondrial length in longitudinal sections, assessing normal animals and 1 day after PT injury (n = 3/group). ON tissue was processed and sectioned as for transverse sections, but sections were cut longitudinally.

**Table 1:** Experimental design for TEM study (transverse sections)

	**Day 1 n animals (mitochondria analysed)**	**Day 7 n animals (mitochondria analysed)**
Normal	4 (344)	
Normal handled	4 (224)	-
PT unhandled	4 (584)	4 (272)
PT handled	4 (398)	4 (306)
PT 670 nm	4 (466)	4 (327)
Total n	36 (2,921)	

Note an additional day 1 cohort was used for longitudinal sections. TEM is transmission electron microscopy. Numbers shown are animals (total number of mitochondria analysed in transverse sections for each treatment or injury group).

### TEM assessment of mitochondrial ultrastructure: image acquisition and analysis

For each ON segment, a single section that clearly contained the injury site was chosen for analysis. TEM assessments were conducted by collecting 30 images for transverse sections and 20 images for longitudinal sections (using separate animal cohorts, as described above) in this single section of ON at the injury site, following a predetermined sampling pattern in the ventral region, similar to that previously described [54] and as illustrated in Figure 1A. Transverse sections were imaged using a Philips CM-10 TEM (Eindhoven, The Netherlands) attached to an Olympus Megaview III camera (1376x1032 pixels), at an accelerating voltage of 80 kV at 25000x magnification, corresponding to a field of view size of 10.4 μm^2^. The total area imaged per section was 312 μm^2^, which corresponds to approximately 0.3% of the ventral ON area, as used in assessments of myelination of axons at the ultrastructural level [54,55]. Longitudinal sections were imaged using a JEOL JEM-2001 (Japan) TEM attached to an Oris SC1000 CCD camera (40008x2672 pixels) at an accelerating voltage of 120 kV, at 4000x magnification, which on this microscope corresponded to a field of view size of 34.2 μm^2^.

All visible mitochondria in all images were analysed (>220 mitochondria/group for transverse sections and >50 mitochondria/group for longitudinal sections). A mitochondrion was defined as a structure displaying a double membrane, being darker (more electron-dense) compared to the surroundings and/or displaying convolutions [56]. Mitochondrial length and the cross-sectional area of each mitochondrial profile (the latter as measured by manually tracing around the outer membrane) were assessed using Image J analysis software (NIH, public domain). To generate a quantitative measure of profile shape, diameter of mitochondrial profiles at the longest extent was measured, followed by a second similar measurement perpendicular to the first and through the midline. These values were used to generate a ratio which reflected how circular or elliptical a mitochondrial profile was, with a ratio of 1.0 denoting a circle, while higher ratios represented increasingly elliptical profiles.

The location of mitochondria within images of transverse sections was defined morphologically as either ‘axonal’ or ‘glial’, the latter of which denoted any mitochondrion not clearly within an axon (Figure 1B). Further, mitochondrial profiles exhibiting features of mitochondrial autophagy [57] were separately quantified. To generate mitochondrial density values, the area of glial processes was measured in every field of view and a sum of these areas was generated per animal. Glial mitochondrial density was derived by dividing the number of mitochondria in glia in all fields of view for each animal by the total area of the glial compartment. Axonal mitochondrial density was calculated similarly, by subtracting the glial areas from the total fields of view to provide the axonal areas. The axonal area therefore includes myelin as well as small spaces between axons.

### Enzyme activity assays: tissue preparation

Activities of the two mitochondrial enzymes, aconitase and citrate synthase, were assessed in ONs from a second cohort of animals. Changes in enzyme activities as a consequence of secondary degeneration were assessed in four groups (n = 6/group): Normal, day 1, day 7 and day 28 after injury. The effect of R/NIR-IT on the activity of these enzymes following injury was evaluated in a separate group of animals at days 1 and 7: at each time point there was a PT handled control group and a PT 670 nm light treatment group (n = 6/group). To collect tissue, following anaesthesia, the ON was accessed as for PT surgery and a 2 mm segment of ON containing the lesion site (or similar area for normal controls) rapidly removed onto a microscope slide on a metal plate over dry ice. The ON segments were dissected into dorsal and ventral halves longitudinally, while viewed under a dissecting microscope, and dorsal or ventral halves pooled for each group, snap frozen in liquid nitrogen and stored at −80°C. The dissection procedure was accomplished in less than 2 min. Thus assays were conducted on a single tissue sample (comprising pooled optic nerve segments from 6 animals) for each experimental group.

### Mitochondrial aconitase activity assay

Mitochondrial aconitase activity was determined using an assay kit (Abcam, Cambridge, UK, ab83459), according to the manufacturer’s instructions. In brief, pooled dorsal or ventral ONs were homogenised in the supplied assay buffer on ice in a glass Dounce homogeniser, homogenates centrifuged at 800 g for 10 mins at 4°C to remove nuclei and gross cellular debris, and the resultant supernatant further centrifuged at 20,000 g for 15 minutes at 4°C in an ultracentrifuge (Optima TLX, Beckman-Coulter) to collect the mitochondrial fraction. The pellets were dissolved into 0.1 ml of cold assay buffer, sonicated for 20 s in a water bath (Branson 3210) and stored at −80°C until assayed. Aconitase activity was determined in triplicate by comparison to a standard curve, and expressed in mU, where one unit of aconitase is the amount of enzyme that will isomerise 1 μmol of citrate to isocitrate per min at pH 7.4 at 25°C.

### Citrate synthase activity assay

After homogenisation on ice in Cell Lytic buffer (Sigma-Aldrich Co., St. Louis, MO) as described above, ON samples were assayed for citrate synthase in triplicate using an assay kit (Sigma, CS0720), according to the manufacturer’s instructions. Citrate synthase activity was expressed in μmol/min. Results for both enzyme assays were expressed/mg protein; based on the pellets of the first centrifugation after homogenisation, due to the limitations of ON homogenate volumes (Thermo Scientific).

### Statistical analyses

Mean mitochondrial densities, areas and shapes, as reflected by the ratio of diameters, were assessed with one-way ANOVA in SPSS (version 20, IBM). Homogeneity of variance was confirmed using Levene’s test. Frequency distributions for mitochondrial area and shape were generated and a modified version of the two-sample Kolmogorov-Smirnov (KS) test was used to determine statistically significant differences between distributions in R stat (version 2.15). The modification of the KS test used a permutation analysis (re-sampling without replacement, repetitions = 5000) to accurately address whether experimental condition was non-randomly associated with the measured distributions, without making assumptions about the nature of the underlying error distribution [58]. Permutations were carried out at the level of animals to avoid pseudo-replication. The permutation approach is based on the assumption that if there is no association between treatment and mitochondrial area/shape, then permuting or randomising this association will, on average, not change the test statistic (d) calculated by the KS test. Statistical significance was then assessed as the probability of obtaining a d-value in the randomised samples that is as or more extreme than the d-value generated by the measured dataset, with p ≤ 0.05 being considered statistically significant. Pair-wise comparisons were limited to three specific questions associated with our hypotheses; specifically, changes with injury only, changes in injured animals following R/NIR-IT, and comparison of treated to normal animals.

Data for observations of mitochondrial autophagic profiles were analysed in the context of a regression analysis, due to the high variability, lack of clear demonstration of normal distributions and the non-factorial study design. We used the same permutation approach as described above, with the test statistic being the F-statistic of the parameter in question. As in a typical regression analysis, the significance of each model term was judged by comparing models that differed by only one term. As such, this analysis incorporated the variation found in all experimental groups and was not limited to pair-wise comparisons. Data for day 1 and day 7 were analysed separately, using the same normal control group for comparison. This analysis was performed in MATLAB^®^ version R2012a (MathWorks).

For the enzyme assays, statistically significant differences between groups were determined by multivariate ANOVA (MANOVA) in SPSS, with activity in dorsal and ventral optic nerve as dependent variables and time point (day 1, day 7, day 28) and treatment (PT handled, PT 670 nm) as between subjects factors, with subsequent follow-up ANOVAs and Bonferroni *post hoc* tests as appropriate. Values for the test statistic Pillai’s trace (V) and follow-up ANOVAs (F) are provided, with p ≤ 0.05 being considered statistically significant.

## Competing interests

The authors declare no competing interests.

## Authors’ contributions

NC, CAB, MA and EB conducted the research. NC, JH analysed the data and conducted statistical assessments. ARH, SAD, MF, NC designed the study and contributed to writing of the manuscript. All authors read and approved the final manuscript.

## References

[B1] Fitzgerald M Bartlett CA Harvey AR Dunlop SA Early events of secondary degeneration after partial optic nerve transection: an immunohistochemical study J Neurotrauma 2010 27 439 452 10.1089/neu.2009.1112 19852581

[B2] Yoles E Schwartz M Degeneration of spared axons following partial white matter lesion: implications for optic nerve neuropathies Exp Neurol 1998 153 1 7 10.1006/exnr.1998.6811 9743562

[B3] McEwen M Sullivan P Rabchevsky A Springer J Targeting mitochondrial function for the treatment of acute spinal cord injury Neurotherapeutics 2011 8 168 179 10.1007/s13311-011-0031-7 21360236 PMC3101832

[B4] Knöferle J Koch JC Ostendorf T Michel U Planchamp V Mechanisms of acute axonal degeneration in the optic nerve in vivo Proc Natl Acad Sci 2010 107 6064 6069 10.1073/pnas.0909794107 20231460 PMC2851885

[B5] Chinopoulos C Adam-Vizi V Mitochondrial Ca2+ sequestration and precipitation revisited FEBS J 2010 277 3637 3651 10.1111/j.1742-4658.2010.07755.x 20659160

[B6] Peng T-I Jou M-J Oxidative stress caused by mitochondrial calcium overload Ann N Y Acad Sci 2010 1201 183 188 10.1111/j.1749-6632.2010.05634.x 20649555

[B7] Camello-Almaraz C Gomez-Pinilla PJ Pozo MJ Camello PJ Mitochondrial reactive oxygen species and Ca2+ signaling Am J Physiol Cell Physiol 2006 291 1082 1088 10.1152/ajpcell.00217.2006 16760264

[B8] Wells J Kilburn MR Shaw JA Bartlett CA Harvey AR Early in vivo changes in calcium ions, oxidative stress markers, and ion channel immunoreactivity following partial injury to the optic nerve J Neurosci Res 2012 90 606 618 10.1002/jnr.22784 22038561

[B9] Twig G Elorza A Molina AJA Mohamed H Wikstrom JD Fission and selective fusion govern mitochondrial segregation and elimination by autophagy EMBO J 2008 27 433 446 10.1038/sj.emboj.7601963 18200046 PMC2234339

[B10] Barsoum MJ Yuan H Gerencser AA Liot G Kushnareva Y Nitric oxide-induced mitochondrial fission is regulated by dynamin-related GTPases in neurons EMBO J 2006 25 3900 3911 10.1038/sj.emboj.7601253 16874299 PMC1553198

[B11] Hom JR Gewandter JS Michael L Sheu S-S Yoon Y Thapsigargin induces biphasic fragmentation of mitochondria through calcium-mediated mitochondrial fission and apoptosis J Cell Physiol 2007 212 498 508 10.1002/jcp.21051 17443673

[B12] Knott AB Perkins G Schwarzenbacher R Bossy-Wetzel E Mitochondrial fragmentation in neurodegeneration Nat Rev Neurosci 2008 9 505 518 18568013 10.1038/nrn2417 PMC2711514

[B13] Fitzgerald M Bartlett CA Evill L Rodger J Harvey AR Secondary degeneration of the optic nerve following partial transection: the benefits of lomerizine Exp Neurol 2009 216 219 230 10.1016/j.expneurol.2008.11.026 19118550

[B14] Fitzgerald M Payne SC Bartlett CA Evill L Harvey AR Secondary retinal ganglion cell death and the neuroprotective effects of the calcium channel blocker lomerizine Invest Ophthalmol Vis Sci 2009 50 5456 5462 10.1167/iovs.09-3717 19474405

[B15] Youle RJ van der Bliek AM Mitochondrial fission, fusion, and stress Science 2012 337 1062 1065 10.1126/science.1219855 22936770 PMC4762028

[B16] Trimmer PA Swerdlow RH Parks JK Keeney P Bennett JP Jr Abnormal mitochondrial morphology in sporadic Parkinson’s and Alzheimer’s disease cybrid cell lines Exp Neurol 2000 162 37 50 10.1006/exnr.2000.7333 10716887

[B17] Gegg ME Cooper JM Chau KY Rojo M Schapira AH Mitofusin 1 and mitofusin 2 are ubiquitinated in a PINK1/parkin-dependent manner upon induction of mitophagy Hum Mol Genet 2010 19 4861 4870 10.1093/hmg/ddq419 20871098 PMC3583518

[B18] Tretter L Adam-Vizi V Inhibition of Krebs cycle enzymes by hydrogen peroxide: A key role of [alpha]-ketoglutarate dehydrogenase in limiting NADH production under oxidative stress J Neurosci 2000 20 8972 8979 11124972 10.1523/JNEUROSCI.20-24-08972.2000 PMC6773008

[B19] Eells JT Henry MM Summerfelt P Wong-Riley MTT Buchmann EV Therapeutic photobiomodulation for methanol-induced retinal toxicity Proc Natl Acad Sci 2003 100 3439 3444 10.1073/pnas.0534746100 12626762 PMC152311

[B20] Rojas JC Lee J John JM Gonzalez-Lima F Neuroprotective effects of near-infrared light in an in vivo model of mitochondrial optic neuropathy J Neurosci 2008 28 13511 13521 10.1523/JNEUROSCI.3457-08.2008 19074024 PMC2637249

[B21] Oron A Oron U Streeter J Taboada LD Alexandrovich A Low-level laser therapy applied transcranially to mice following traumatic brain injury significantly reduces long-term neurological deficits J Neurotrauma 2007 24 651 656 10.1089/neu.2006.0198 17439348

[B22] Stemer AB Huisa BN Zivin JA The evolution of transcranial laser therapy for acute ischemic stroke, including a pooled analysis of NEST-1 and NEST-2 Curr Cardiol Rep 2010 12 29 33 10.1007/s11886-009-0071-3 20425181 PMC2821619

[B23] Karu T Primary and secondary mechanisms of action of visible to near-IR radiation on cells J Photochem Photobiol B 1999 49 1 17 10.1016/S1011-1344(98)00219-X 10365442

[B24] Wong-Riley MTT Liang HL Eells JT Chance B Henry MM Photobiomodulation directly benefits primary neurons functionally inactivated by toxins J Biol Chem 2005 280 4761 4771 15557336 10.1074/jbc.M409650200

[B25] Karu TI Mitochondrial signaling in mammalian cells activated by red and near-IR radiation Photochem Photobiol 2008 84 1091 1099 10.1111/j.1751-1097.2008.00394.x 18651871

[B26] Fitzgerald M Bartlett CA Payne SC Hart NS Rodger J Near infrared light reduces oxidative stress and preserves function in CNS tissue vulnerable to secondary degeneration following partial transection of the optic nerve J Neurotrauma 2010 27 2107 2119 10.1089/neu.2010.1426 20822460

[B27] Hogan V White K Edgar J McGill A Karim S Increase in mitochondrial density within axons and supporting cells in response to demyelination in the Plp1 mouse model J Neurosci Res 2009 87 452 459 10.1002/jnr.21867 18803300

[B28] Perge JA Koch K Miller R Sterling P Balasubramanian V How the optic nerve allocates space, energy capacity, and information J Neurosci 2009 29 7917 7928 10.1523/JNEUROSCI.5200-08.2009 19535603 PMC2928227

[B29] Chakrabarti L Eng J Ivanov N Garden G La Spada A Autophagy activation and enhanced mitophagy characterize the Purkinje cells of pcd mice prior to neuronal death Mol Brain 2009 2 24 10.1186/1756-6606-2-24 19640278 PMC2729476

[B30] Nixon RA Wegiel J Kumar A Yu WH Peterhoff C Extensive involvement of autophagy in Alzheimer disease: an immuno-electron microscopy study J Neuropathol Exp Neurol 2005 64 113 122 15751225 10.1093/jnen/64.2.113

[B31] Ashrafi G Schwarz TL The pathways of mitophagy for quality control and clearance of mitochondria Cell Death Differ 2013 20 31 42 10.1038/cdd.2012.81 22743996 PMC3524633

[B32] Klionsky DJ Abeliovich H Agostinis P Agrawal DK Aliev G Guidelines for the use and interpretation of assays for monitoring autophagy in higher eukaryotes Autophagy 2008 4 151 175 18188003 10.4161/auto.5338 PMC2654259

[B33] Castro L Rodriguez M Radi R Aconitase is readily inactivated by peroxynitrite, but not by its precursor, nitric oxide J Biol Chem 1994 269 29409 29415 7961920

[B34] Prabakaran S Swatton J Ryan M Huffaker S Huang JTJ Mitochondrial dysfunction in schizophrenia: evidence for compromised brain metabolism and oxidative stress Mol Psychiatry 2004 9 684 697 15098003 10.1038/sj.mp.4001511

[B35] Hargreaves I Duncan A Wu L Agrawal A Land J Inhibition of mitochondrial complex IV leads to secondary loss complex II–III activity: implications for the pathogenesis and treatment of mitochondrial encephalomyopathies Mitochondrion 2007 7 284 287 10.1016/j.mito.2007.02.001 17395552

[B36] Chepelev NL Bennitz JD Wright JS Smith JC Willmore WG Oxidative modification of citrate synthase by peroxyl radicals and protection with novel antioxidants J Enzyme Inhib Med Chem 2009 24 1319 1331 10.3109/14756360902852586 19795928

[B37] Hall ED Vaishnav RA Mustafa AG Antioxidant therapies for traumatic brain injury Neurotherapeutics 2010 7 51 61 10.1016/j.nurt.2009.10.021 20129497 PMC2818465

[B38] Ott M Gogvadze V Orrenius S Zhivotovsky B Mitochondria, oxidative stress and cell death Apoptosis 2007 12 913 922 10.1007/s10495-007-0756-2 17453160

[B39] Zhang S-J Sandström ME Lanner JT Thorell A Westerblad H Activation of aconitase in mouse fast-twitch skeletal muscle during contraction-mediated oxidative stress Am J Physiol Cell Physiol 2007 293 C1154 C1159 10.1152/ajpcell.00110.2007 17615160

[B40] Natoli R Zhu Y Valter K Bisti S Eells J Gene and noncoding RNA regulation underlying photoreceptor protection: microarray study of dietary antioxidant saffron and photobiomodulation in rat retina Mol Vis 1801 2010 16 PMC2932490 20844572

[B41] Frank S Dysregulation of mitochondrial fusion and fission: an emerging concept in neurodegeneration Acta Neuropathol 2006 111 93 100 10.1007/s00401-005-0002-3 16468021

[B42] Rintoul GL Reynolds IJ Mitochondrial trafficking and morphology in neuronal injuryhim Biophys Acta 2010 1802 143 150 10.1016/j.bbadis.2009.09.005 19747973

[B43] Manteifel V Bakeeva L Karu T Ultrastructural changes in chondriome of human lymphocytes after irradiation with He·Ne laser: Appearance of giant mitochondria Photochem Photobiol B 1997 38 25 30 10.1016/S1011-1344(96)07426-X 9134752

[B44] Vanneste J Bosch de Aguilar P Mitochondrial alterations in the spinal ganglion neurons in ageing rats Acta Neuropathol 1981 54 83 87 10.1007/BF00691335 6263035

[B45] Manteifel VM Karu TI Structure of mitochondria and activity of their respiratory chain in successive generations of yeast cells exposed to He-Ne laser light Biol Bull 2005 32 556 566 10.1007/s10525-005-0143-x 16535977

[B46] Tretter L Takacs K Kövér K Adam-Vizi V Stimulation of H2O2 generation by calcium in brain mitochondria respiring on α-glycerophosphate J Neurosci Res 2007 85 3471 3479 10.1002/jnr.21405 17600838

[B47] De Vos KJ Allan VJ Grierson AJ Sheetz MP Mitochondrial function and actin regulate dynamin-related protein 1-dependent mitochondrial fission Curr Biol 2005 15 678 683 10.1016/j.cub.2005.02.064 15823542

[B48] Koopman WJH Verkaart S Visch H-J van der Westhuizen FH Murphy MP Inhibition of complex I of the electron transport chain causes O2 − ·-mediated mitochondrial outgrowth Am J Physiol Cell Physiol 2005 288 C1440 C1450 10.1152/ajpcell.00607.2004 15647387

[B49] Anesti V Scorrano L The relationship between mitochondrial shape and function and the cytoskeleton Biochim Biophys Acta 2006 1757 692 699 10.1016/j.bbabio.2006.04.013 16729962

[B50] Kokkinopoulos I Colman A Hogg C Heckenlively J Jeffery G Age-related retinal inflammation is reduced by 670 nm light via increased mitochondrial membrane potential Neurobiol Aging 2012 Epub 2012 May 16 10.1016/j.neurobiolaging.2012.04.014 22595370

[B51] Perkins GA Ellisman MH Fox DA Three-dimensional analysis of mouse rod and cone mitochondrial cristae architecture: bioenergetic and functional implications Mol Vis 2003 9 60 73 12632036

[B52] Levkovitch-Verbin H Quigley HA Martin KR Zack DJ Pease ME A model to study differences between primary and secondary degeneration of retinal ganglion cells in rats by partial optic nerve transection Invest Ophthalmol Vis Sci 2003 44 3388 3393 10.1167/iovs.02-0646 12882786

[B53] Brondon P Stadler I Lanzafame RJ A study of the effects of phototherapy dose interval on photobiomodulation of cell cultures Lasers Surg Med 2005 36 409 413 10.1002/lsm.20183 15880587

[B54] Payne SC Bartlett CA Harvey AR Dunlop SA Fitzgerald M Chronic swelling and abnormal myelination during secondary degeneration after partial injury to a central nervous system tract J Neurotrauma 2011 28 1077 1088 10.1089/neu.2010.1665 21381867

[B55] Payne SC Bartlett CA Harvey AR Dunlop SA Fitzgerald M Myelin sheath decompaction, axon swelling, and functional loss during chronic secondary degeneration in rat optic nerve Invest Ophthalmol Vis Sci 2012 53 6093 6101 10.1167/iovs.12-10080 22879411

[B56] Ju W-K Kim K-Y Lindsey JD Angert M Duong-Polk KX intraocular pressure elevation induces mitochondrial fission and triggers OPA1 release in glaucomatous optic nerve Invest Ophthalmol Vis Sci 2008 49 4903 4911 10.1167/iovs.07-1661 18469184 PMC2688012

[B57] Ding WX Yin XM Mitophagy: mechanisms, pathophysiological roles, and analysis Biol Chem 2012 393 547 564 22944659 10.1515/hsz-2012-0119 PMC3630798

[B58] Randomization M Bootstrap and Monte Carlo Methods in Biology 2007 London: Chapman and Hall

